# Infectious Dose of *Listeria monocytogenes* in Outbreak Linked to Ice Cream, United States, 2015

**DOI:** 10.3201/eid2212.160165

**Published:** 2016-12

**Authors:** Régis Pouillot, Karl C. Klontz, Yi Chen, Laurel S. Burall, Dumitru Macarisin, Matthew Doyle, Kären M. Bally, Errol Strain, Atin R. Datta, Thomas S. Hammack, Jane M. Van Doren

**Affiliations:** Food and Drug Administration, College Park, Maryland, USA (R. Pouillot, K.C. Klontz, Y. Chen, D. Macarisin, M. Doyle, E. Strain, T.S. Hammack, J.M. Van Doren);; Food and Drug Administration, Laurel, Maryland, USA (L.S. Burall, A.R. Datta);; Via Christi Hospitals, Wichita, Kansas, USA (K.M. Bally)

**Keywords:** Listeria monocytogenes, dose-response, infectious dose, outbreak investigation, United States, bacteria, foodborne illness, ice cream, food safety

## Abstract

The relationship between the number of ingested *Listeria monocytogenes* cells in food and the likelihood of developing listeriosis is not well understood. Data from an outbreak of listeriosis linked to milkshakes made from ice cream produced in 1 factory showed that contaminated products were distributed widely to the public without any reported cases, except for 4 cases of severe illness in persons who were highly susceptible. The ingestion of high doses of *L. monocytogenes* by these patients infected through milkshakes was unlikely if possible additional contamination associated with the preparation of the milkshake is ruled out. This outbreak illustrated that the vast majority of the population did not become ill after ingesting a low level of *L. monocytogenes* but raises the question of listeriosis cases in highly susceptible persons after distribution of low-level contaminated products that did not support the growth of this pathogen.

## Abstract 2

Listeriosis can occur in susceptible populations when products with low-level contamination are distributed widely.

Understanding the likelihood of developing invasive listeriosis after ingesting a given number of *Listeria monocytogenes* cells (dose-response relationship) is important in managing risks linked to this pathogen in food. Nevertheless, several challenges hamper characterization of this dose-response relationship, including the lack of an appropriate animal model, the relative rarity of outbreaks, long incubation periods that impede the collection of well-preserved implicated food samples, and heterogeneity of the initial contamination level (*1*).

In early 2015, an outbreak of invasive listeriosis linked to ice cream products was identified in the United States (*2*). A total of 10 case-patients with listeriosis related to this outbreak were reported from Arizona and Oklahoma (1 case each); Texas (3 cases); and Kansas (5 cases, all in inpatients of 1 hospital) (*2*). *L. monocytogenes* isolates from 4 of the Kansas case-patients were indistinguishable by pulsed-field gel electrophoresis from isolates recovered from ice cream made in 1 plant of the implicated company (factory 1). The isolate from the fifth Kansas case-patient did not match any isolate recovered in this outbreak investigation. *L. monocytogenes* isolates from patients in other states were linked to ice cream products manufactured in another facility (factory 2) of the same company (*2*). The US Food and Drug Administration (FDA) collected a large volume of ice cream from factory 1 for microbiological testing.

This outbreak provided a unique opportunity to assess exposure levels to *L. monocytogenes* from implicated ice cream products among infected persons and the overall population. Because ice cream has a long shelf life and *L. monocytogenes* does not grow but survives for long periods in frozen products (*3*), the level of *L. monocytogenes* in implicated products manufactured during the outbreak, although collected after the outbreak, was likely to be representative of levels in products eaten by exposed persons. We assessed the outbreak data to gain insight into contamination levels among products from 1 factory implicated in the outbreak, the number of *L. monocytogenes* cells ingested by specific subpopulations during this outbreak, and the dose-response relationship for *L. monocytogenes*.

## Materials and Methods

### Framework for Dose-Response Derivation

In microbial dose-response frameworks, it is generally assumed that as few as 1 independently acting cell that survives host defense measures can initiate infection (1-hit theory [*4**,**5*]). This minimal infective dose of 1 cell is associated with a probability (*r*) of infection. Assuming *r* is low and constant within a subpopulation (Technical Appendix), *r* can be estimated by the ratio of the number of invasive listeriosis cases in a subpopulation (*X*_p_), by the estimated number of *L. monocytogenes* cells ingested by the subpopulation *D_p_*; that is, *r* = *X_p_* / *D_p_*. In addition to using this classical derivation of *r,* we estimated in this study *r* values using the *L. monocytogenes* dose-response model of Pouillot et al. (*6*) (Technical Appendix).

### Listeriosis Cases

This study considers only the 4 hospitalized Kansas case-patients whose illnesses were confirmed to be linked to ingestion of products manufactured in factory 1. Illness onset dates ranged from January 2014 through January 2015 (Figure). All 4 were >67 years and <84 years of age. Medical records review indicated all 4 had underlying medical conditions that contributed to compromised immune function before exposure to *L. monocytogenes* in milkshakes. Food histories were available for 3 of the Kansas case-patients. All patients with food histories ate product 1 from factory 1 through milkshakes. One patient had 2 milkshakes (1 day at lunch and the following day at dinner); another had 2 milkshakes (1 day at dinner and 6 days later at dinner), and the remaining patient had 3 milkshakes (1 day at dinner and 4 and 9 days later at dinner and lunch, respectively). Two serving units of product 1, each weighing ≈80 g, were used to prepare each milkshake. Strains of *L. monocytogenes* isolated from the 4 patients were indistinguishable by pulsed-field gel electrophoresis to strains recovered from product 1.

**Figure:**
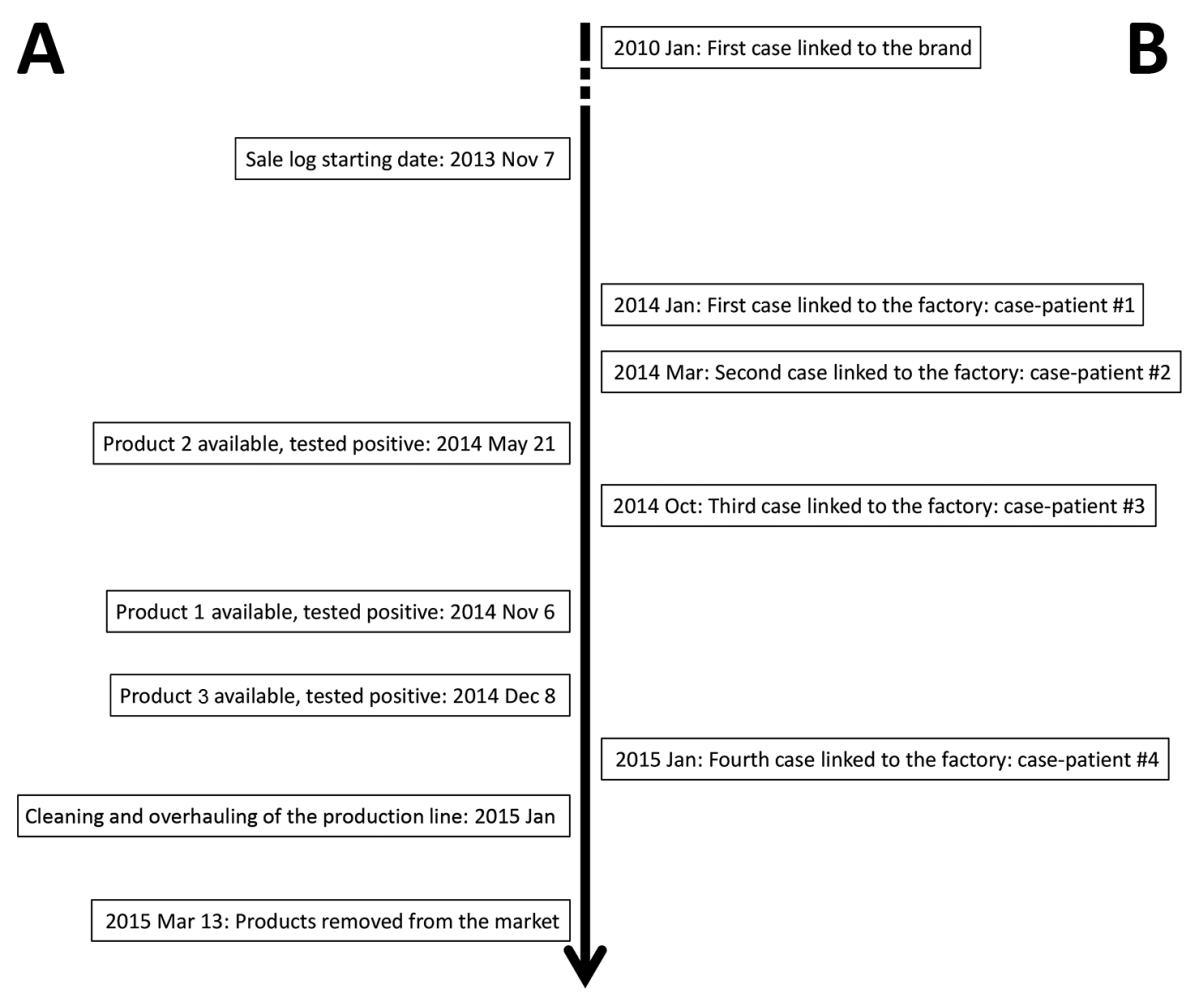
Timeline of listeriosis outbreak linked to ice cream, United States, 2015. A) Data for products produced in factory 1 (*2*); B) data for outbreak start and 4 case-patients at 1 hospital in Kansas.

### Number of *L. monocytogenes* Cells Ingested by the Population

The factory 1 production line linked to the Kansas cases made 8 different types of ice cream products (products 1–8) (*7*). (The website for this reference identifies 10 universal product codes corresponding to 8 different types of ice cream products; 2 products were sold individually and grouped in larger packages). FDA collected and counted *L. monocytogenes* cells in samples of products 1–3 (*8*; L.S. Burall, unpub. data). We characterized the variability of *L. monocytogenes* levels in products 1–3 (Technical Appendix).

No samples of products 4–8 were collected. In a low-exposure scenario, products that were not tested were assumed to be uncontaminated. In a medium-exposure scenario and in a high- exposure scenario, contamination levels were predicted on the basis of the processes used to produce these products. Specifically, we specified in these scenarios that contamination levels were similar for products 1 and 4 and were similar for products 2 and 5–8 because the process used to produce product 4 was similar to that used for product 1, whereas production processes for products 5–8 were similar to that for product 2.

The number of *L. monocytogenes* cells ingested by the population was then estimated by multiplying the average number of *L. monocytogenes* organisms per serving by the number of servings distributed in the various subpopulations. The number of ice cream servings distributed in the various subpopulations was estimated from product distribution records for factory 1.

We do not know when contamination of the production line at factory 1 began. We isolated *L. monocytogenes* from a product manufactured on this line on May 21, 2014, but we had no samples manufactured before this date. Although the first known case associated with the brand of ice cream occurred in January 2010, the first case-patient specifically linked to factory 1 was hospitalized in Kansas on December 24, 2013, and listeriosis was diagnosed in January 2014 (patient 1, Figure). In the low-exposure scenario and medium-exposure scenario, we assumed the date at which contamination began at factory 1 was December 1, 2013, that is, a few weeks before hospitalization of the first case-patient whose illness was linked to ice cream produced at this facility. Contamination could have begun earlier than this date given that 1 listeriosis case-patient whose illness was linked to the same brand, but produced at factory 2, became ill in 2010. In the high-exposure scenario, we assumed contamination began 2.5 years before the outbreak was recognized, that is, midway between 2010 and the date the outbreak was recognized.

To estimate the proportion of servings that reached inpatients deemed to be highly susceptible to listeriosis, we multiplied the proportion of ice cream distributed to hospitals for patient consumption by the overall proportion of intensive care unit (ICU) beds in these hospitals (i.e., 10%) as a surrogate of the proportion of inpatients deemed to be highly susceptible to invasive listeriosis. To estimate the proportion of servings potentially eaten by pregnant women, ≥ 65 y. and ≥ 75 y. persons, we assumed that the implicated brand was eaten by different subpopulations similarly to other brands of ice cream (Technical Appendix).

To understand why 4 cases of ice cream–associated listeriosis clustered at a single hospital, we created 2 indices for the hospitals that received contaminated product(s) from factory 1 at least 1 time during November 7, 2013–March 16, 2015. The first index ascertained the severity of patient illness at each hospital (illness score) and was calculated by determining the percentage of total beds constituting ICU beds (scale: 0%–4.9%, 1 point; 5%–9.9%, 2 points; 10%–14.9%, 3 points; and >15%, 4 points). Hospitals were contacted by telephone and queried about the total number of beds licensed and the number dedicated to treatment of patients in ICU (medical, surgical, pediatric, neonatal, and burn). To quantify the availability of contaminated products at each hospital (supply score), we divided the total number of servings shipped to each facility during the recorded distribution period (16 months) by the total number of hospital beds (scale: <1 serving per bed, 1 point; 1–3.99, 2 points; 4–6.99, 3 points; and >7, 4 points). Using the 2 indices, we summed scores for all hospitals (maximum possible score 8) as an overall measure of patient illness and potential product exposure.

## Results

### Number of *L. monocytogenes* Cells per Serving

All tested samples of product 1 manufactured before the outbreak was recognized were positive for *L. monocytogenes* (*8*). Assuming the 5 lots of product 1 tested were representative of all lots of contaminated product 1, we estimated the mean number of *L. monocytogenes* cells in each 80-g unit of product 1 at 620 CFU (95% credible interval [CrI] 380–2,100 CFU). From the distribution of contamination level inferred from the model, we estimated that 0.1% of servings of product 1 had a dose >7,400 CFU (95% CrI 4,400–58,000 CFU) (see Table 1 for other statistics). *L. monocytogenes* was recovered from 80% of 294 units of product 2 (unit size 70 g) tested (mean 310 CFU/serving [95% CrI 55–11,000 CFU/serving]). Of the 95 units of product 3 tested, 45% yielded *L. monocytogenes* (mean 0.12 CFU/g).

**Table 1:** Estimated contamination level of *Listeria monocytogenes* per gram and per serving unit of 3 products in a multistate outbreak of ice cream–associated listeriosis, United States, 2015

Product/dose	Estimate (95% credible interval)			Quantile (95% credible interval)			
	Mean	SD		90%	99%	99.9%	99.99%
Product 1							
Per g	8 (5–26)	10 (6–62)		17 (10–60)	46 (27–270)	92 (55–730)	160 (97–1,500)
Per 80-g serving	620 (380–2,100)	760 (460–4,900)		1,300 (820–4,800)	3,700 (2,200–22,000)	7,400 (4,400–58,000)	13,000 (7,800–120,000)
Product 2							
Per g	5 (1–160)	200 (17–35,000)		2 (1–10)	48 (11–620)	520 (91–12,000)	3,600 (470–140,000)
Per 70-g serving	310 (55–11,000)	14,000 (1,200–2,500,000)		140 (43–710)	3,400 (800–43,000)	37,000 (6,400–840,000)	250,000 (33,000–9,800,000)
Product 3							
Per g	0.12 in 45% of products						
Per 160-g serving	8.64 in 45% of servings						

### Number of *L. monocytogenes* Cells Consumed by the Population

Sales data suggested widespread distribution of contaminated products to hospitals and the general population (e.g., schools, grocery stores, restaurants). We estimated that the general population ingested a total of 1.5 × 10^9^ (low-exposure scenario) to 1.4 × 10^10^ (high-exposure scenario) *L. monocytogenes* cells (Table 2). We estimated that, overall, the highly susceptible population ingested 7.2 × 10^6^ (low-exposure scenario) to 3.3 × 10^7^ (high-exposure scenario) *L. monocytogenes* cells.

**Table 2:** Probability of invasive listeriosis after ingestion of ice cream products contaminated with *Listeria monocytogenes*, United States, 2015

Exposure scenario/model	Population, no. cases in population				
	All, n = 4	Highly susceptible, n = 4	Pregnant, n = 0*	Age >65 y, n = 4	Age >75 y, n = 2
Lower†					
*r* Constant					
No. *L. monocytogenes* cells consumed	1.5 × 10^9^	7.2 × 10^6^	2.2 × 10^7^	2.3 × 10^8^	1.2 × 10^8^
Estimated *r* parameter	2.6 × 10^−9^	5.5 × 10^−7^	<2.3 × 10^−8^	1.7 × 10^−8^	1.7 × 10^−8^
Corresponding to 1 case every … servings‡	37,867	181	>4,363	5,756	5,832
Log_10_(*r*) normally distributed					
Estimated *μ* parameter	−9.38	−6.19	<(−7.92)	−8.00	−8.02
Estimated *σ* parameter	0.88	0.24	0.54	0.54	0.54
Medium§					
*r* Constant					
No. *L. monocytogenes* cells consumed	6.2 × 10^9^	1.5 × 10^7^	8.9 × 10^7^	9.4 × 10^8^	4.8 × 10^8^
Estimated *r* parameter	6.5 × 10^−10^	2.7 × 10^−7^	<5.6 × 10^−9^	4.3 × 10^−9^	4.2 × 10^−9^
Corresponding to 1 case every … servings‡	154,612	375	>17,812	23,501	23,811
Log_10_(*r*) normally distributed					
Estimated *μ* parameter	−10.0	−6.40	<(−8.49)	−8.60	−8.62
Estimated *σ* parameter	0.88	0.24	0.54	0.54	0.54
High¶					
*r* Constant					
No. *L. monocytogenes* cells consumed	1.4 × 10^10^	3.3 × 10^7^	2.0 × 10^8^	2.1 × 10^9^	1.0 × 10^9^
Estimated *r* parameter	2.9 × 10^−10^	1.2 × 10^−7^	<2.6 × 10^−9^	1.9 × 10^−9^	1.9 × 10^−9^
Corresponding to 1 case every … servings‡	339,153	816	>39,071	51,552	52,230
Log_10_(*r*) normally distributed					
Estimated *μ* parameter	−10.3	−6.80	<(−8.83)	−8.97	−8.97
Estimated *σ* parameter	0.88	0.24	0.54	0.54	0.54

*0.5 used for computation. †Products 1–3 contaminated beginning 2013 Dec 1; products 4–8 not contaminated. ‡Corresponding to 1 case every … servings, Including 10,000 *L. monocytogenes* cells. §Products 1–8 contaminated beginning 2013 Dec 1. ¶Products 1–8 contaminated beginning 2012 Jun 1.

Among hospitals that received >1 products from the production line of factory 1 known to produce contaminated ice cream, the median percentage of total beds constituting ICU beds (severity of illness score) was 8.7% (range 0%–70.7%; mean 10%). The median number of servings per bed (supply score) over the recorded distribution period (16 months) was 2 (range 0.1–93.7; mean 4.3). The Kansas hospital with the 4 cases of ice cream–associated listeriosis had 62.2 servings of the implicated products per bed (13.5% of beds in the hospital were ICU beds); the servings per bed value for the hospital was exceeded by only 1 other hospital (93.7 servings/bed; 6.5% ICU beds). After combining the severity of illness and supply scores for each hospital, we found the median value was 5 (range 2–7; mean 4.6); a combined score of 7 was achieved by 9% of hospitals, of which 1 was the Kansas hospital with the 4 cases (the hospital with 93.7 servings/bed had a combined score of 6).

### Probability of Infection after Ingestion of 1 Cell

Under the low-exposure scenario, we estimated that the probability of infection, *r*, after ingestion of 1 bacterium in the overall population was

Using this same approach, we determined the value of *r* for the overall population was *r* = 6.5 × 10^−10^ under the medium-exposure scenario and *r* = 2.9 × 10^−10^ under the high-exposure scenario (Table 2). The integration of the model by Pouillot et al. (*6*), considering a normal distribution of the log_10_ of the *r* parameter in the population rather than a constant one, led to a distribution with a mean −9.38 and an SD of 0.88 for the overall population under the lower-exposure scenario, a mean of −10.0 for the medium-exposure scenario, and a mean of −10.3 for the high-exposure scenario (Table 2).

We also assessed persons at greatest risk for invasive listeriosis, including pregnant women, highly susceptible persons (e.g., those with compromised immune function), persons >65 years of age, and persons >75 years of age (Table 2). Because no ice cream–associated cases were reported among pregnant women, we used an estimate of 0.5 cases and provided only an upper limit value for *r*. (This value was chosen arbitrarily. A Poisson process with mean 0.5 would have led to 0 cases in 90% of occurrence.)

## Discussion

This outbreak investigation provided unique data to characterize the dose-response relationship between *L. monocytogenes* in general and susceptible populations. Multiple factors compelled us to estimate as precisely as possible doses of *L. monocytogenes* ingested by consumers of contaminated products. First, the number of samples microbiologically tested was by far the largest ever reported from an outbreak setting (*8*). Second, because ice cream preserves the viability of *L. monocytogenes* but does not support its growth, levels of contamination were likely to have been accurately measured and have remained relatively constant over the extended shelf lives of the products. Finally, an exceptionally stable level of contamination within product types minimized variability in exposures. Hospital records indicated that patient 4 drank milkshakes made with product 1 on 3 different days during January 11–19, 2015, before sepsis caused by *L. monocytogenes* infection was diagnosed on January 23. This patient could have eaten ice cream from lots we enumerated. Only 4 (0.2%) of 2,320 samples of product 1 yielded a concentration >100 CFU/g, equivalent to a dose of >16,000 *L. monocytogenes* cells per milkshake (2 servings of 80 g × 100 CFU/g, assuming the 2 servings were >100 CFU/g). Inferences on the interlot, interbox, and intrabox variability helped us define precisely the distribution of contamination levels from serving to serving and confirmed that a very high concentration of *L. monocytogenes* cells in any given serving unit was not likely. The estimated mean dose per milkshake is 1,240 *L. monocytogenes* cells (95% CrI 760–4,200 *L. monocytogenes* cells). We estimate that 1 of 10,000 milkshakes would have a load >26,000 *L. monocytogenes* cells (95% CrI 15,600–240,000 *L. monocytogenes* cells). Assuming there was no initial contamination of the milkshake machines and no growth of the pathogen in the milkshakes, the mean contamination level of *L. monocytogenes* in the milkshakes (8 cells/g of ice cream) was relatively low compared with contamination levels in some other outbreaks (*9**–**12*). However, in the absence of leftovers from the actual implicated milkshakes, we cannot rule out the possibility that the 4 susceptible patients received some of the highest contaminated products from the factory line, triggering infection. Experimental trials of *L. monocytogenes* growth in milkshakes made from these naturally contaminated ice cream samples held at room temperature showed an absence of growth during 8 hours and an average population level increase after 14 hours limited to 1.14 log CFU/g (*13*). We cannot exclude the possibility that variations in procedures used to clean the milkshake machines might have enabled isolated microbial growth on >1 machines. We believe the extremely high prevalence of contamination of product 1 might have inoculated >1 machines with repeated preparations over the long period during which contaminated products were distributed; however, no *Listeria* was isolated from samples collected from these machines after the outbreak was recognized (Charles Hunt, Kansas Department of Health and Environment, pers. comm.).

Although the 4 cases of ice cream–associated listeriosis in a single hospital raise the possibility of a systematic problem within the hospital, it is also possible that the combination of severely ill patients, including some with specific risk factors for listeriosis such as hematologic cancers (*14*), in a setting in which a large amount of contaminated ice cream was served contributed to this series of infections. Medical staff at the hospital also might have had a heightened suspicion of listeriosis after diagnosis of the initial case, which might have increased the likelihood of detecting cases. Overall, the Kansas hospital received 55% of all product 1 sold to hospitals. Thus, observing the 4 cases in this specific hospital was not improbable. (The probability to observe 4 successes out of 4 trials is 9% when the independent probability of success is 55%.)

Although precise quantification of exposure to *L. monocytogenes* ingestion through contaminated ice cream is difficult to infer for specific persons, an assessment of exposures among populations is more feasible. Despite the relatively low levels of contamination of ice cream products in this listeriosis outbreak, the exceptionally high prevalence of contaminated products, combined with the protracted duration of contamination of the production line (at least 1 year and possibly longer), contributed to exposure of many persons to *L. monocytogenes*. This finding suggests that widespread distribution of contaminated products with low-dose contamination by *L. monocytogenes* in a product that does not support growth of *L. monocytogenes* might lead to only a limited number of reported infections. We focused our study on 1 cluster of outbreak-related cases, the one for which FDA was able to collect samples of ice cream for microbiological testing. Five other cases of ice cream–associated invasive listeriosis were identified in states other than Kansas; these cases were linked to another production factory operated by the same company, expanding further the quantity of contaminated ice cream sold to the public.

The Food and Agriculture Organization of the World Health Organization (FAO/WHO) (*15*) estimated a *r* parameter of 3.2 × 10^−7^ in a well-documented listeriosis outbreak involving immunocompromised patients in Finland in 1998–1999 (*16**,**17*); in this outbreak, the median estimated dose ingested was 8.2 × 10^3^ *L. monocytogenes*. Our estimate of the *r* parameter for the susceptible population is in the same order of magnitude (1.2 × 10^−7^ to 5.5 × 10^−7^). In the population of pregnant women, FAO/WHO (*15*) estimated a *r* parameter of 2.6 × 10^−11^ on the basis of an outbreak of cheese-associated listeriosis involving pregnant Hispanic women in Los Angeles County, California, USA, in 1985 in which the estimated dose was 1.7 × 10^7^ *L. monocytogenes* (*10*). More recently, Imanishi et al. (*18*) estimated an attack rate of 1 case/10,000 exposed pregnant women in Colorado, USA, during a 2011 multistate outbreak of listeriosis linked to contaminated cantaloupe (*19*); no enumeration data were available in this outbreak. Studies have shown that cut cantaloupe supports the growth of *L. monocytogenes* (*20**,**21*), suggesting that some exposures could have been high during this outbreak. In the ice cream–associated outbreak described here, no cases were reported among pregnant women despite presumably widespread exposures among this subgroup of susceptible persons. Specifically, a large number of contaminated ice cream products were presumably ingested by pregnant women during the long duration of contamination of the production line. From the expected number of *L. monocytogenes* cells ingested by this subpopulation, we estimate, under the various assumptions used in this study, a value of *r* <2.6 × 10^−9^ to *r* <2.3 × 10^−8^. In summary, estimates for *r* derived in the present study are comparable in order of magnitude with estimates derived from previous outbreaks, a finding that is noteworthy in light of the low levels of contamination of ice cream products and the fact that these products did not support growth. Although other outbreaks were linked to higher level of contamination per serving than in the present study, the number of contaminated servings was much lower in those outbreaks than in the present one.

On the other hand, estimates for *r* obtained in the present study are higher than those estimated by using epidemiologic data (*6**,**15**,**17*). Using epidemiologic data, FAO/WHO (*15*) estimated that the probability of infection after consumption of 1 *L. monocytogenes* cell is in the order of *r =* 5 × 10^−12^ for susceptible persons (immunocompromised persons, pregnant women, and elderly persons), and 5 × 10^−14^ for nonsusceptible persons (*15*). These values predict the occurrence of 1 listeriosis case for every 20 million exposures to 10,000 *L. monocytogenes* cells in the susceptible population (10,000, which was chosen arbitrarily, would correspond to the dose after ingestion of 100 g of a product contaminated at 100 CFU/g) and 1 case of listeriosis for every 2 billion exposures to 10,000 *L. monocytogenes* cells in the nonsusceptible population. The estimates obtained in our study were much higher than these values: 1 case expected for every 339,200 servings of 10,000 bacteria per serving, such as for the general population in the high-exposure scenario. Similarly, using the model of Pouillot et al. (*6*), we estimated that values from the ice cream outbreak data are ≈2 log_10_ higher than those based on epidemiologic data. A possible explanation for these differences is that a particularly virulent strain of *L. monocytogenes* was present in ice cream. Differences in *r* estimates obtained from outbreak investigations versus epidemiologic data also could result from observation bias, wherein recognition of cases instigates a study, leading to high number of cases for equation input and thus higher estimates for *r*. In contrast, situations where contaminated products are distributed but no cases are recognized are underrepresented in such evaluations.

This outbreak of ice cream–associated listeriosis recognized in 2015 demonstrates that illnesses can occur when products with low-level contamination that do not support growth are distributed widely to the public, even though it is not possible to conclude with certainty whether the cases were linked directly to the products or indirectly after a growth step on a milkshake machine. The outbreak also illustrates that even when the distribution of products contaminated with *L. monocytogenes* is widespread, most consumers of the products will not become ill when contamination levels are low and no growth is facilitated. Finally, this outbreak adds yet further evidence of the risk for listeriosis faced by persons with weakened immune systems and calls for effective risk management to mitigate infections (*22*).

Technical Appendix Framework for dose-response in a study of the infectious dose of *Listeria monocytogenes* in an outbreak linked to ice cream, United States, 2015; derivation of the contamination level distributions; and estimation of the proportion of ice cream eaten by various subpopulations.
