# Supplementary material for: Infectious Dose of Listeria monocytogenes in Outbreak Linked to Ice Cream, United States, 2015
Source: Emerg Infect Dis. 2016 Dec;22(12):2113–9. doi: 10.3201/eid2212.160165 (PMC5189132; doi:10.3201/eid2212.160165)
Supplement: Technical Appendix — Framework for dose-response in a study of the infectious dose of Listeria monocytogenes in an outbreak linked to ice cream, United States, 2015; derivation of the contamination level distributions; and estimation of the proportion of ice cream eaten by various subpopulations. [file 16-0165-Techapp-s1.pdf]

Article DOI: http://dx.doi.org/10.3201/eid2212.160165

Infectious Dose of *Listeria monocytogenes* in Outbreak Linked to Ice Cream, United States, 2015

Technical Appendix

Framework for Dose-Response

Mathematically, the resulting data from an exposed population can be represented as infection indicator x_i for each individual, which take a value of 1 if infection is observed and a value of 0 otherwise (*1*). For a serving i, under the 1-hit theory assumptions (*2,3*) we have

$$x_i \sim Bernoulli(\pi_i) \text{ with } \pi_i = 1 - (1 - r_i)^{d_i}$$

where π_i is the probability that the individual got infected after ingesting a specific serving i, d_i is the dose (number of *Listeria monocytogenes* cells) ingested through the serving i and r_i is the average probability that a pathogen from serving i survive the host-pathogen response and initiate infection (*1*). For the population, the observed number of cases is a realization of all those independent Bernoulli processes. We have:

(Eq. 1) $E(X) = \sum_{i=1}^{m}(1 - (1 - r_i)^{d_i})$

where, additionally, E(X) is the expected number of cases and m is the number of exposed persons. The characterization of the dose-response thus requires the knowledge of the probability that the pathogen survive the host-pathogen response (r_i, variable, from serving to serving, notably because of individual susceptibility) and the individual doses (d_i, variable, from serving to serving), considered as independent from r_i. Some authors reported that the r parameter could be considered as constant within a given subpopulation (e.g., susceptible and nonsusceptible subpopulation within [*4*]). Under this assumption, (Eq. 1) simplifies to

(Eq. 2) $E\left(X_p\right) = \sum_{i=1}^{m_p}(1 - (1 - r)^{d_i})$.

where X_p is the number of cases and m_p is the number of persons in the subpopulation p and the distribution of the dose only has to be known. Moreover, because r is usually very small for *L. monocytogenes,* $1-(1-r)^d \approx rd$. Under this limit, the equation simplifies to

(Eq. 3) $E(X_p) = \sum_{i=1}^{m_p} rd_i = r \times m_p \times \bar{d}$

where $\bar{d}$ is the mean number dose ingested in the population. From (Eq. 3), r can be then evaluated as

(Eq. 4) $\hat{r} = \frac{X_p}{m_p \hat{\bar{d}}} = \frac{X_p}{\widehat{D_p}}$,

where $\widehat{D_p}$ is the estimated number of *L. monocytogenes* ingested by the population.

To better characterize variability in *L. monocytogenes* dose-response, Pouillot et al. (*5*) used a log-normal distribution to describe r_i, rather than a constant, that is $\log_{10}(r_i) \sim \text{normal}(\mu_p, \sigma_p)$, with negligible probability that r >1. During an outbreak, σ_p characterize only the within subpopulation variability in susceptibility because strain virulence variability can be neglected (*5*,*6*). Following assumptions used in Pouillot et al. (*5*) and Food and Drug Administration/Food Safety and Inspection Service (*6*), we considered a high variability in susceptibility within the overall population (90% of the individual variability in r may be contained within a range of 2.9 $\log_{10}$, leading to $\sigma = 0.82 \log_{10}$), a medium variability in susceptibility within the pregnant women population and the older adult populations (90% of the individual variability in r may be contained within a range of 1.8 $\log_{10}$, leading to $\sigma = 0.55 \log_{10}$), and a low variability in susceptibility in the highly susceptible population (90% of the individual variability in r may be contained within a range of 0.8 $\log_{10}$, leading to $\sigma = 0.24 \log_{10}$). With an assumption of a log-normal distribution of r_i, (Eq. 1) cannot be simplified and the equation should be integrated numerically over the distribution of r_i and d_i. Integrations were performed using R software (*7*).

Derivation of the Contamination Level Distributions

Briefly, 2,320 samples of ice cream product 1 (80 g each), 295 samples of product 2 (70 g), and 96 samples of product 3 (160 g) were microbiologically tested. *L. monocytogenes* cells were enumerated in these products by using the most probable number (MPN) method from

dilution assay results. Microbiological methods and summary statistics are described in Chen et al, (*8*) and Burall et al (unpub. data).

Product 1 samples were collected from 7 lots. All tested products from the 5 first lots were contaminated (2,020 positive samples of 2,020 tested). After the first reports of contaminated products, the production line was reportedly cleaned and overhauled in factory 1 on January 30, 2015 (9). Ninety-six percent (287 positive samples of 300) of products tested from 2 later lots, manufactured after the cleaning, were contaminated. *L. monocytogenes* contamination levels were extremely homogeneous among products within boxes, boxes within lots, and across lots (*8*). The observed mean concentration of *L. monocytogenes* in product 1 before the cleaning of the line was 9 MPN/g of product.

From the experimental design (*8*) and from the raw results, we characterize in this study the variability in *L. monocytogenes* levels across lots (lot to lot; data from 5 lots), across boxes within a lot (box to box; 8–53 boxes tested per lot), and across servings within a box (serving to serving; 10–20 servings tested per box). We restricted the analysis to lots manufactured before cleaning and overhauling of the production line. To evaluate the lot-to-lot, box-to-box within lots, and serving-to-serving within box variability, a hierarchical Bayesian framework was developed based on the results of the 2,020 dilution assays conducted on product 1. The model is written $\lambda_l \sim \text{Normal}(\mu, \sigma_1)$; $\lambda_{b,l} \sim \text{Normal}(\lambda_l, \sigma_2)$; $\lambda_{s,b,l} \sim \text{Normal}(\lambda_{b,l}, \sigma_3)$; $\boldsymbol{p}_{s,b,l} \sim \text{Binomial}\left(\boldsymbol{n_s}, 1 - \exp\left(-10^{\boldsymbol{v_s}\lambda_{s,b,l}}\right)\right)$; where *l* stands for lot, *b* stands for box, and *s* stands for serving, *p*, *n*, and *v* are the number of positive tubes, the number of tested tubes and the "grams" of tested product for each tubes in the dilution assay experiment. Uninformative prior distributions $\mu \sim \text{Normal}(-4, 10)$ and $\sigma_x^{-2} \sim \text{Uniform}(0, 10)$ were used.

Product 2 samples were collected from 10 lots manufactured before the cleaning of the manufacturing line. Both the prevalence of contamination and the number of *L. monocytogenes* cells detected in product 2 samples were lower and more variable than respective values for product 1 (L.S. Burall et al., unpub. data). The prevalence was estimated to be 80% (234/294 samples, L.S. Burall et al., unpub. data). The observed overall mean contamination (using a value of one half the limit of quantification, that is, 0.016 MPN/g for negative samples) for product 2 was 3.4 MPN/g. We derived a Bayesian model similar to the one for product 1 to evaluate variability in *L. monocytogenes* levels across lots (lot-to-lot; data from 10 lots), across

boxes within a lot (box-to-box; 1 to 6 boxes tested per lot), and across servings within a box (serving-to-serving; 5–10 servings tested per box).

Ninety-five samples of product 3 from 5 lots manufactured before the cleaning of the manufacturing line were tested. Forty-three (45%) were positive for *L. monocytogenes*. The mean *L. monocytogenes* contamination level for positive samples was lower, estimated to 0.12 MPN/g and the standard deviation to 0.14 MPN/g).

For product 1, from the Bayesian model, the mean of the $\log_{10}$ concentrations is estimated 0.70 $\log_{10}$ CFU/g, with an interlot variability of 0.21 $\log_{10}$ CFU/g, an interbox variability of 0.14 $\log_{10}$ CFU/g, and an intrabox variability of 0.33 $\log_{10}$ CFU/g (Technical Appendix Table 1). The lot-to-lot variation is not known as precisely as the other levels of variability because fewer lots than boxes or products were examined.

From these results, we simulated the production of the manufacturing line using the empirical posterior distributions from the Bayesian analysis (Technical Appendix Figure). Under the model (assuming that the 5 lots are representative of all lots), Table 1 in the main document reports estimations for different quantiles of the distributions.

From the Bayesian model, the mean of the *L. monocytogenes* $\log_{10}$ concentrations in product 2 in the lots examined was estimated as –1.43 $\log_{10}$/g, with an interlot variability of 0.38, an interbox variability of 0.99, and an intrabox variability of 0.82 (Technical Appendix Table 1). The credible interval are much larger than those obtained for product 1, reflecting the wider variability and the lower number of tested samples. The mean dose for one 70-g serving of product 2 (the serving size of this product) was estimated as 310 cfu (95% credible interval [CrI] 55–11,000 CFU). Table 1 in the main text provides additional estimates for various quantiles in the distribution.

Because the number of tested samples for product 3 was low (n = 95), we did not derive a distribution for this product but considered that, as observed in the tested sample, 45% of these product 3 were contaminated and that the average concentration of *L. monocytogenes* in contaminated products 3 was 0.12 *L. monocytogenes* cells per gram.

In further estimation of the prevalence of contaminated products, 100% will be used for product 1 and product 1 like, 80% for product 2 and product 2 like, and 45% for product 3.

Estimation of the Proportion of Ice Cream Eaten by Various Subpopulations

Demographic data were estimated from data provided by the Centers for Disease Control and Prevention (*10*) and by the US Census Bureau (*11*). Per capita consumption of ice cream for pregnant women and for the overall population were estimated by using the FARE software (Food Analysis and Residue Evaluation, v.11.10, leased from Exponent, Inc, Washington, DC, USA.) from the 1999–2012 National Health and Nutrition Examination Survey/What We Eat In America (NHANES/WWEIA) (*12*), considering any ice cream consumption the first day of survey (Technical Appendix Table 2). Comparable estimates were done for persons ≥65 years and ≥75 years of age (Technical Appendix Table 2).

References

1. Schmidt PJ, Pintar KD, Fazil AM, Topp E. Harnessing the theoretical foundations of the exponential and beta-Poisson dose-response models to quantify parameter uncertainty using Markov chain Monte Carlo. Risk Anal. 2013;33:1677–93. PubMed http://dx.doi.org/10.1111/risa.12006

2. Haas CN, Rose JB, Gerba CP. Quantitative microbial risk assessment. New York: Wiley; 1999.

3. Teunis PF, Havelaar AH. The beta Poisson dose-response model is not a single-hit model. Risk Anal. 2000;20:513–20. PubMed http://dx.doi.org/10.1111/0272-4332.204048

4. Food and Agriculture Organization/World Health Organization. Risk assessment of *Listeria monocytogenes* in ready to eat foods. Technical report. Report no.: Microbiological Risk Assessment Series 5. Rome: The Organizations; 2004 [cited 2016 Jan 28]. ftp://ftp.fao.org/docrep/fao/010/y5394e/y5394e.pdf

5. Pouillot R, Hoelzer K, Chen Y, Dennis SB. *Listeria monocytogenes* dose response revisited—incorporating adjustments for variability in strain virulence and host susceptibility. Risk Anal. 2015;35:90–108. PubMed http://dx.doi.org/10.1111/risa.12235

6. Food and Drug Administration. Quantitative assessment of relative risk to public health from foodborne *Listeria monocytogenes* among selected categories of ready-to-eat foods [cited 2016 Jan 28]. http://www.fda.gov/downloads/Food/FoodScienceResearch/UCM197330.pdf

7. R Development Core Team. R: a language and environment for statistical computing. 2008 [cited 2012 Mar 20]. http://www.R-project.org

8. Chen Y, Burall L, Macarisin D, Pouillot R, Strain E, De Jesus A, et al. Enumeration of *Listeria monocytogenes* in ice cream products linked to a multistate listeriosis outbreak. J Food Prot. In press 2016.

9. Centers for Disease Control and Prevention. Multistate outbreak of listeriosis linked to Blue Bell Creameries products (final update). 2015 [cited 2015 Dec 10]. http://www.cdc.gov/listeria/outbreaks/ice-cream-03-15/

10. Curtin SC, Abma JC, Ventura SJ, Henshaw SK. Pregnancy rates for U.S. women continue to drop. NCHS Data Brief. 2013;(136):1–8. PubMed

11. US Census Bureau. Annual estimates of the resident population for the United States, regions, states, and Puerto Rico: April 1, 2000 to July 1, 2009. 2015 [cited 2016 Jan 28]. https://www.census.gov/popest/data/state/totals/2009/tables/NST-EST2009-01.xls

12. US Department of Agriculture and US Department of Health and Human Services. What we eat in America [cited 2016 Jan 29]. https://www.ars.usda.gov/northeast-area/beltsville-md/beltsville-human-nutrition-research-center/food-surveys-research-group/docs/wweianhanes-overview/

Technical Appendix Table 1. Results from the Bayesian analysis of the contamination variability for product 1 (2020 MPN results) and Product 2 (294 MPN results)*

Parameter	Symbol	Product 1, mean (95% CrI)	Product 2, mean (95% CrI)
Overall mean of the $\log_{10}$ concentration ($\log_{10}$ MPN/g)	μ	0.70 (0.46–0.91)	−1.43 (−2.03 to −0.89]
Interlot standard deviation	σ_1	0.21 (0.09–0.53)	0.38 (0.02–1.25)
Interbox standard deviation	σ_2	0.14 (0.11–0.16)	0.99 (0.68–1.45)
Interproduct standard deviation	σ_3	0.33 (0.32–0.35)	0.82 (0.74–0.91)

*CrI, credible interval; MPN, most probable number.

Technical Appendix Table 2. Estimation of the proportion of ice cream eaten by various subpopulations*

Variable	United States	Pregnant women	≥65 y	≥75 y	Source
Population size	307,006,550†	3,513,205‡	39,570,590†	18,778,523†	(*10,11*)
Per capita estimate of ice cream eaten per day	20.62 g	25.98 g	24.29 g	25.82 g	1999–2012 NHANES/WWEIA (*12*)
Proportion of ice cream eaten by the specific population§	100%	1.44%	15.2%	7.7%	

*NHANES, National Health and Nutrition Examination Survey; WWEIA, What We Eat in America.
†On July 1, 2009.
‡Evaluated from (*10*) as (4,131,000 births in 2009 in the United States × [9 mo of 30 d] + 1,152,000 abortions in 2009 in the United States × [2 mo of 30 d] + 1,087,000 fetal losses in 2009 in the United States × [3 mo of 30 d]) / 365
§Evaluated as (population in subgroup × per capita consumption for that subgroup)/(total US population × per capita consumption in the United States).

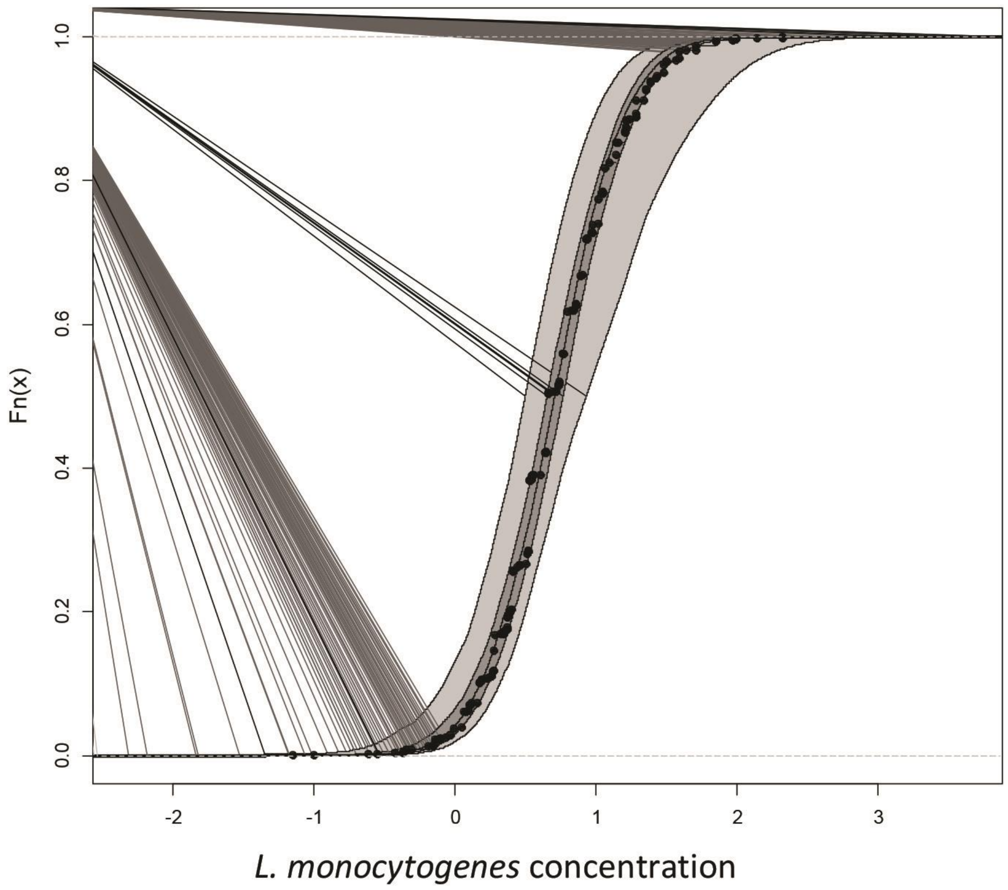

Technical Appendix Figure. Simulated and observed (points) cumulative density function of the *Listeria monocytogenes* contamination for product 1 associated with a listeriosis outbreak, United States, 2015. The envelopes represent the 95% uncertainty interval (light gray) and the 50% uncertainty interval (dark gray).
